# Genome-wide association study of abnormal elevation of ALT in patients exposed to atabecestat

**DOI:** 10.1186/s12864-023-09625-6

**Published:** 2023-09-01

**Authors:** Qingqin S. Li, Stephan Francke, Jan Snoeys, John Thipphawong, Gary Romano, Gerald P. Novak

**Affiliations:** 1 Neuroscience, Janssen Research & Development, LLC, Titusville, NJ 08560 USA; 2 JRD Data Science, Janssen Research & Development, LLC, Titusville, NJ 08560 USA; 3 Computational Science Translational Platforms, Janssen Research & Development, LLC, Spring House, PA 19477 USA; 4 Translational Pharmacokinetics Pharmacodynamics and Investigative Toxicology, Janssen Research & Development, Beerse, Belgium; 5 Present Address: Pharmacogenomics & Biomarker in Clinical Development, Cary, NC USA; 6 Present Address: Passage Bio, Philadelphia, PA USA

**Keywords:** GWAS, ALT elevation, Atabecestat

## Abstract

**Background:**

Atabecestat, a potent brain penetrable BACE1 inhibitor that reduces CSF amyloid beta (Aβ), was developed as an oral treatment for Alzheimer’s disease (AD). Elevated liver enzyme adverse events were reported in three studies although only one case met Hy’s law criteria to predict serious hepatotoxicity.

**Method:**

We performed a case-control genome-wide association study (GWAS) to identify genetic risk variants associated with liver enzyme elevation using 42 cases with alanine transaminase (ALT) above three times the upper limit of normal (ULN) and 141 controls below ULN. Additionally, we performed a GWAS using continuous maximal ALT/ULN (expressed as times the ULN) upon exposure to atabecestat as the outcome measure (n = 285).

**Results:**

No variant passed the genome-wide significance threshold (*p* = 5 × 10^− 8^) in the case-control GWAS. We identified suggestive association signals in genes (*NLRP1*, *SCIMP*, and *C1QBP*) implicated in the inflammatory processes. Among the genes implicated by position mapping using variants suggestively associated (*p* < 1 × 10^− 5^) with ALT elevation case-control status, gene sets involved in innate immune response (adjusted p-value = 0.05) and regulation of cytokine production (adjusted p-value = 0.04) were enriched. One genomic region in the intronic region of *GABRG3* passed the genome-wide significance threshold in the continuous max(ALT/ULN) GWAS, and this variant was nominally associated with ALT elevation case status (*p* = 0.009).

**Conclusion:**

The suggestive GWAS signals in the case-control GWAS analysis suggest the potential role of inflammation in atabecestat-induced liver enzyme elevation.

**Supplementary Information:**

The online version contains supplementary material available at 10.1186/s12864-023-09625-6.

## Background

Idiosyncratic drug-induced liver injury (DILI) is a challenge in drug development. It is both a challenge and an opportunity in the field of pharmacogenomics as genetic variants are often discovered in smaller sample sizes, compared to disease genetics. It is a challenge as the clinical development of compounds may be stopped for safety concerns before the sample size required for genetic discovery is reached. Even if causal genetic variants are identified, they may not have the necessary positive predictive value needed for a clinical application, as DILI is typically a rare event [1].

The discovered genetic risk factors for idiosyncratic DILI are primarily concentrated on polymorphisms in the human leukocyte antigen (HLA) region including HLA-DQB1*06:02 and HLA-A*02:01 associated with amoxicillin-clavulanate induced hepatotoxicity [2], HLA-DRB1*16:01-DQB1*05:02 for flupirtine-induced hepatotoxicity [3], HLA-B*57:01 for flucloxacillin-induced hepatotoxicity [4], HLA-DRB1*1501-HLA-DQB1*0602-HLA-DRB5*0101-HLA-DQA1*0102 for lumiracoxib-induced hepatotoxicity [5], HLA-DRB1*07:01 for lapatinib-induced hepatotoxicity [6], and HLA-DRB1*07 and HLA-DQA1*02 for ximelagatran-induced hepatotoxicity [7]. Additionally, variants in *ST6GAL1*, which plays a role in the systemic inflammatory response, and variants in the intron of *FAM65B*, which play roles in liver inflammation, have been associated with flucloxacillin [4] and antituberculosis drug-induced hepatotoxicity [8], respectively. Polymorphisms from transporters and metabolizing enzymes may also play a role [1].

A guidance for the management of suspected DILI has recommended consideration be given to stopping the suspected drug in any of the following situations: when ALT exceeding 8X the upper limit of normal (ULN); ALT exceeding 5XULN for more than 2 weeks; ALT exceeding 3XULN with total bilirubin > 2XULN or international normalized ratio (INR) > 1.5 (Hy’s Law criteria [9–12]]; ALT exceeding 3XULN with symptoms including fatigue, nausea, vomiting, right upper quadrant pain or tenderness, fever, rash, or eosinophilia [13]. The Roussel UCLAF Causality Assessment Method (RUCAM) may also be applied to assess causality taking age, gender, and medical history into account. In the context of drug development, Hy’s law is more widely used for predicting serious hepatotoxicity [9–12, 14]. The mechanisms of DILI have been reviewed previously and mitochondrial dysfunction, drug-induced free radical production, depletion of glutathione, peroxidation, perturbation of bile flow and activation of innate immune response [1, 15, 16] were implicated.

Atabecestat, a potent brain penetrable BACE1 inhibitor that reduces CSF amyloid beta (Aβ), was developed by Janssen Research & Development LLC and Shionogi and Co Ltd as an oral treatment for Alzheimer’s disease (AD). Elevated liver enzyme (> 3x ULN) adverse events were reported in approximately 10% of participants in three studies [17, 18] although only one case met the Hy’s law criteria to predict serious hepatotoxicity and was described earlier in a case report [19]. Previous challenging/cloning experiments using atabecestat and its metabolites (diaminothiazine [DIAT] and N-acetyl DIAT etc.) implicated the involvement of adaptive immunity in the observed liver enzyme elevation [20]. We herein performed genome wide association study (GWAS) to identify genetic risk variants associated with liver enzyme elevation in cohorts of patients from three phase II clinical studies exposed to atabecestat [17, 18]. Two outcome definitions [dichotomous trait, ALT elevation case control status and a continuous trait [i.e. maximal ALT (max(ALT/ULN)] level any time after drug exposure)] were used in the GWAS.

## Results

**Fig. 1:**
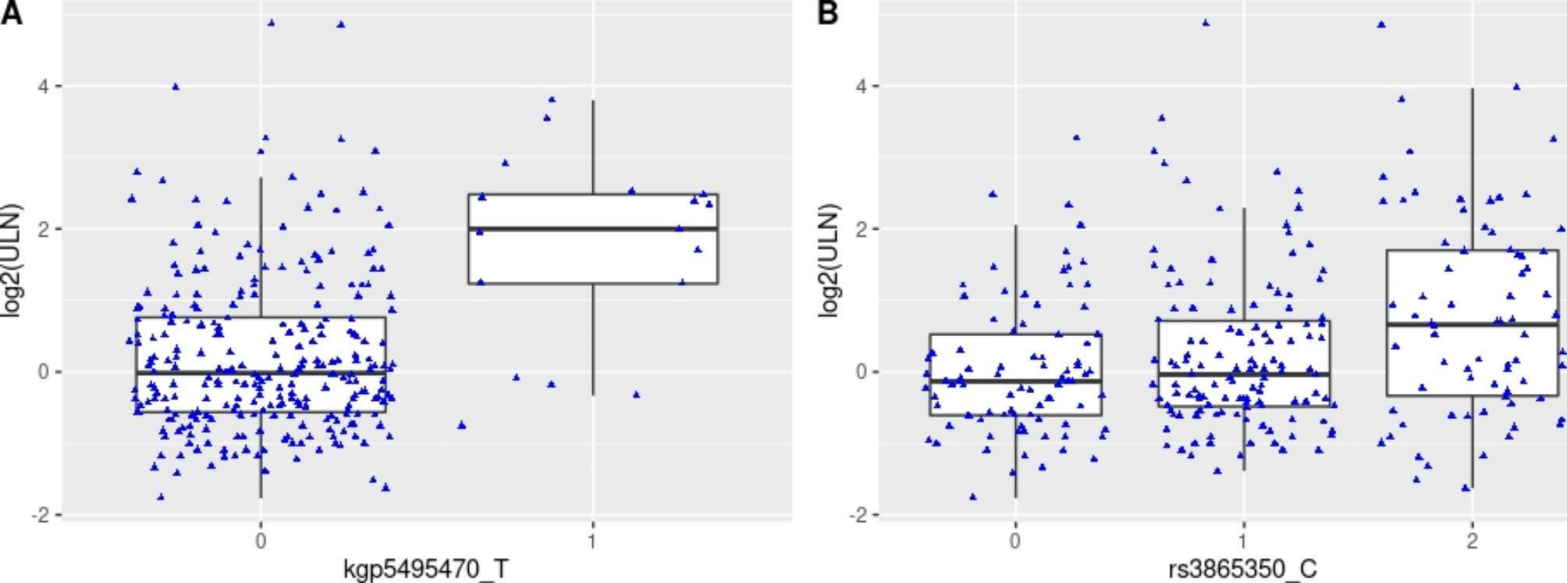
Top variants associated with maximal ALT level. (**A**) kgp5495470/rs114579373 (**B**) rs3865350

The demographic characteristics of patients in the case control cohort is described in Supplemental Table 1. The top variant rs3865350 (Table 1, p = 4.63 × 10^− 7^, Manhattan and QQ plots in Supplemental Figs. 1 and 2, and genotype cluster plot in Supplemental Fig. 3) associated with ALT elevation was from a genomic region on chromosome 17p13.2, annotated to genes *DERL2*, *MIS12*, and *NLRP1*. The *NLRP1* intronic variant kgp8586964 (also known as rs8067359), that was in linkage disequilibrium (LD) with rs3865350 (chr17: 5,381,867) and an eQTL variant for NLRP1 in fibroblast cells, was nominally associated with ALT elevation case control status (Table 1; Fig. 1B, p = 3.02 × 10^− 6^). The G allele of kgp8586964 is associated with greater risk of having ALT elevation (OR = 3.4, 95% confidence interval [2.0, 5.8]), and is also associated with greater level of *NLRP1* expression in fibroblast cell (*p* = 2.2 × 10^− 12^, Supplemental Fig. 4, Data Source: GTEx Analysis Release V8).

**Table 1:** Variants associated with ALT elevation case-control analysis (p-value < 1 × 10^− 5^)

SNP	CHR	BP	A1	A2	F_A	F_U	OR	SE	L95	U95	P	Gene	annot	dist
rs818165	2	10,613,284	A	G	0.1905	0.4574	0.2791	0.3025	0.1543	0.5049	7.30 × 10^− 6^	*AC007249.3*	intergenic	17,606
												*NOL10*	intergenic	97,608
rs12188529	5	16,529,754	A	G	0.2143	0.4929	0.2806	0.2914	0.1585	0.4967	4.82 × 10^− 6^	*FAM134B*	intronic	0
rs3865350	17	5,381,867	C	T	0.7143	0.4007	3.739	0.2704	2.201	6.352	4.63 × 10^− 7^	*DERL2*	intronic	0
rs2309399	17	5,391,690	G	A	0.7143	0.4043	3.684	0.2703	2.169	6.258	8.40 × 10^− 7^	*MIS12*	UTR5	0
kgp8586964	17	5,409,097	G	T	0.7024	0.4071	3.436	0.2679	2.033	5.809	3.02 × 10^− 6^	*NLRP1*	intronic	0

CHR: Chromosome; SNP: SNP ID; BP: Physical position (base-pair)in reference to genome build 37; A1: Minor allele name (based on whole sample); F_A: Frequency of A1 allele in cases; F_U: Frequency of A1 allele in controls; A2: Major allele name; P: Exact p-value; OR: Estimated odds ratio (for A1); L95: Lower bound of 95% confidence interval for odds ratio; U95: Upper bound of 95% confidence interval for odds ratio

**Fig. 2:**
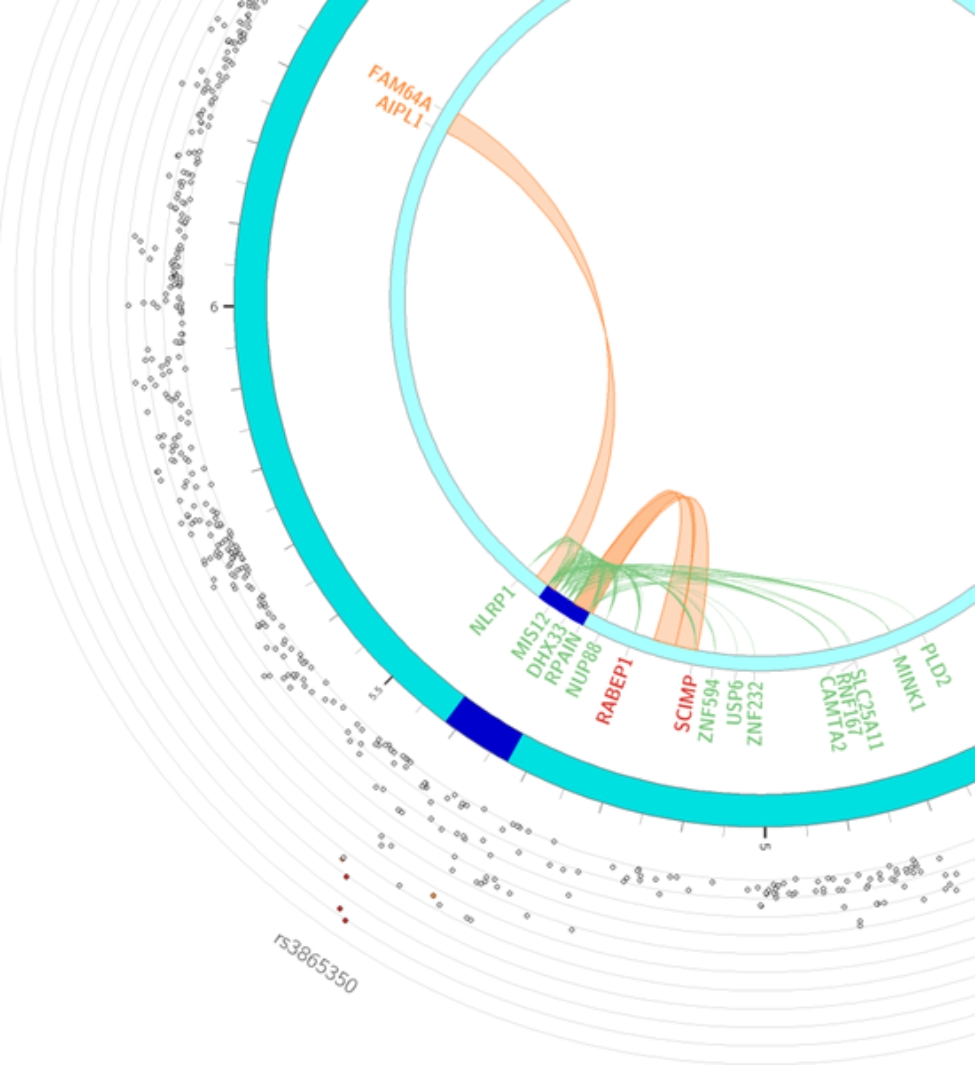
Circos plot for rs3865350 highlighting eQTLs and/or chromatin interactions. The circos plot was generated via FUMA [43] v1.3.4b (https://fuma.ctglab.nl/), the outer most layer is Manhattan plot and the middle layer highlights genomic risk loci (as defined by FUMA [43] using minimum P-value of lead SNPs of 1 × 10^− 5^ and default values for other parameters) in blue, while the inner most layer highlights eQTLs and/or chromatin interactions. Only SNPs with *p* < 0.05 are displayed in the outer ring. SNPs in genomic risk loci are color-coded as a function of their maximum r [2] to the one of the independent significant SNPs in the locus. The rsID of the top SNPs in each risk locus are displayed in the most outer layer. For the inner most layer, if the gene is mapped only by chromatin interactions or only by eQTLs, it is colored orange or green, respectively. It is colored red when the gene is mapped by both

The genomic region (chr17: 5,320,001–5,360,000) forms a long-range 3D chromatin interaction loop with a genomic region (chr17: 5,120,001–5,160,000) containing *SCIMP* (chr17: 5,112,215-5,138,155) encoding SLP adaptor and CSK interacting membrane protein in liver (Source: HiC dataset GSE87112 [21], Fig. 2, Supplemental Table 2). Both *NLRP1* and *SCIMP* are involved in inflammatory processes. rs3865350 was also suggestively associated with max(ALT/ULN) upon exposure to atabecestat (*p* = 5.87 × 10^− 5^). A list of variants associated with ALT elevation case status with a Fisher’s exact test association p-value less than 1 × 10^− 5^ is available in Supplemental Table 3 A together with association test results from continuous trait max(ALT/ULN) and sensitivity analyses including a logistic regression model for the ALT elevation case-control status (*p* = 1.55 × 10^− 5^ for rs3865350) and the similar GWAS analyses in the subsets of samples of European ancestry (*p* = 1.84 × 10^− 5^ for case-control analysis using 39 cases and 127 controls; *p* = 5.60 × 10^− 5^ for the continuous trait analysis in a cohort of 258 samples).

Two variants in the intronic region of GABRG3 on chromosome 15 passed the genome-wide significance threshold in the max(ALT/ULN) single-variant analyses (rs188010617- *p* = 3.04 × 10^− 9^ and rs193168063 - *p* = 1.18 × 10^− 8^). Rs188010617 was also nominally associated with ALT elevation case-control status (*p* = 0.01) in the logistic regression sensitivity analysis (this variant was not tested in Fisher’s exact test as it was not directly genotyped).

A list of variants associated with max(ALT/ULN) with an association p-value less than 1 × 10^− 6^ is available in Supplemental Table 3B together with association test results from Fisher’s exact and other sensitivity analyses. One of the top variants associated in the continuous maximal ALT trait analysis was kgp5495470 (also known as rs114579373) on chromosome 5q34 [*p* = 2.19 × 10^− 7^ for max(ALT/ULN), Manhattan and QQ plots in Supplemental Figs. 1 and 2, and genotype cluster plot in Supplemental Fig. 3]. Using the dichotomized ALT elevation trait, the same variant was associated with the case/control status (*p* = 3.23 × 10^− 5^, Fig. 1A). Eleven out of 84 (13%) chromosomes in cases vs. 4 out of 282 (1%) chromosomes in controls carried the T allele.

No individual gene passed the genome-wide significance threshold in any of the gene-based analyses (multi-marker analysis of genomic annotation (MAGMA) gene-based analysis). *GSTM4* was nominally associated with case control ALT elevation status (*p* = 0.003). A full list of genes with nominal association p-value less than 0.005 is available in Supplemental Table 4.

No gene set passed the study-wide significance threshold in any of the gene set-based analyses. Gene sets involved in immune signaling pathways such as regulation of IFNa signaling (*p* = 1.25 × 10^− 5^), type I interferon pathway (*p* = 1.66 × 10^− 5^), IL27 pathway (*p* = 3.22 × 10^− 5^), IL10 pathway (*p* = 0.0002), alpha/beta T-cell activation (*p* = 0.0003) were nominally associated with ALT elevation case control status (Table 2, a full list of gene sets with nominal association p-value less than 0.005 is available in Supplemental Table 5). In addition, genes involved in the glutathione metabolism pathway were nominally associated with ALT elevation (*p* = 0.002). Gene sets involved in lectin pathway of complement activation was nominally associated with max(ALT/ULN) (*p* = 1.23 × 10^− 5^) upon exposure to atabecestat.

**Table 2:** Pathways enriched in ALT elevation case control status GWAS in the MAGMA analysis

NGENES	BETA	BETA_STD	SE	P	FULL_NAME
12	1.2331	0.033236	0.29242	1.25 × 10^− 5^	Curated_gene_sets:reactome_regulation_of_ifna_signaling
7	1.4693	0.030251	0.35395	1.66 × 10^− 5^	Curated_gene_sets:st_type_i_interferon_pathway
24	0.80854	0.030808	0.20227	3.22 × 10^− 5^	Curated_gene_sets:pid_il27_pathway
10	0.92136	0.022671	0.24383	7.91 × 10^− 5^	Curated_gene_sets:reactome_synthesis_of_substrates_in_n_glycan_biosythesis
16	0.89253	0.027774	0.25252	0.00020489	Curated_gene_sets:biocarta_il10_pathway
62	0.44334	0.02712	0.13012	0.00032907	GO_bp:go_regulation_of_alpha_beta_t_cell_activation
15	0.95853	0.028882	0.28255	0.00034726	Curated_gene_sets:biocarta_il22bp_pathway
23	0.68542	0.025567	0.20322	0.00037292	GO_bp:go_positive_regulation_of_activated_t_cell_proliferation
10	1.0675	0.026266	0.32388	0.00049164	Curated_gene_sets:reactome_il_6_signaling
13	0.83744	0.023492	0.26035	0.00064987	Curated_gene_sets:ding_lung_cancer_mutated_frequently

Among the genes implicated by position mapping, using variants suggestively associated with ALT elevation case control status (p < 1 × 10^− 5^), gene sets involved in innate immune response (adjusted p-value = 0.05) and regulation of cytokine production (adjusted p-value = 0.04) were enriched, among those in the GO [22] biological processes (from MsigDB [23] c5; Supplemental Table 6). *NLRP1* and *C1QBP* were the genes responsible for these pathway enrichments.

## Discussion

We performed GWAS using two phenotype definitions to identify genetic variants that might explain ALT elevation observed in a subset of patients exposed to atabecestat. Even though we did not identify any variant or gene passing the genome wide significance threshold associated with case-control status, several genes and pathways implicated in the immune response and glutathione metabolism pathway surfaced as candidate signals explaining the drug-induced ALT elevation. There was a genome wide significant finding in the continuous trait max(ALT/ULN) GWAS, with nominal support (*p* = 0.01) from the case-control status analysis.

Among the top variants associated with ALT elevation case control status, three genes implicated in the inflammatory process were implicated. *NLRP1* (NLR family pyrin domain containing 1) was implicated by position mapping and encodes a member of the Ced-4 family of apoptosis proteins. Ced-family members contain a caspase recruitment domain (CARD) and are known to be key mediators of programmed cell death. NLRP1 contains a distinct N-terminal pyrin domain (PYD), which may mediate protein-protein interactions. NLRP1 and other leucine-rich repeat (LRR)-containing protein (NLR) family members can initiate the formation of inflammasomes which are cytosolic multi-protein complexes serving as sensors for infection and damage to illicit inflammatory response [24, 25]. It has been reported that inflammasome is activated in liver of cholestatic patients and aggravates hepatic injury in bile duct-ligated mouse [26]. *SCIMP* was implicated by long range chromatin interaction. *SCIMP* encodes a transmembrane adaptor protein that is expressed in antigen-presenting cells and localized in the immunologic synapse and is involved in major histocompatibility complex class II signal transduction and immune synapse formation, and mediates TLR-4 mediated cytokine responses in macrophages upon LPS stimulation [27].

**Fig. 3:**
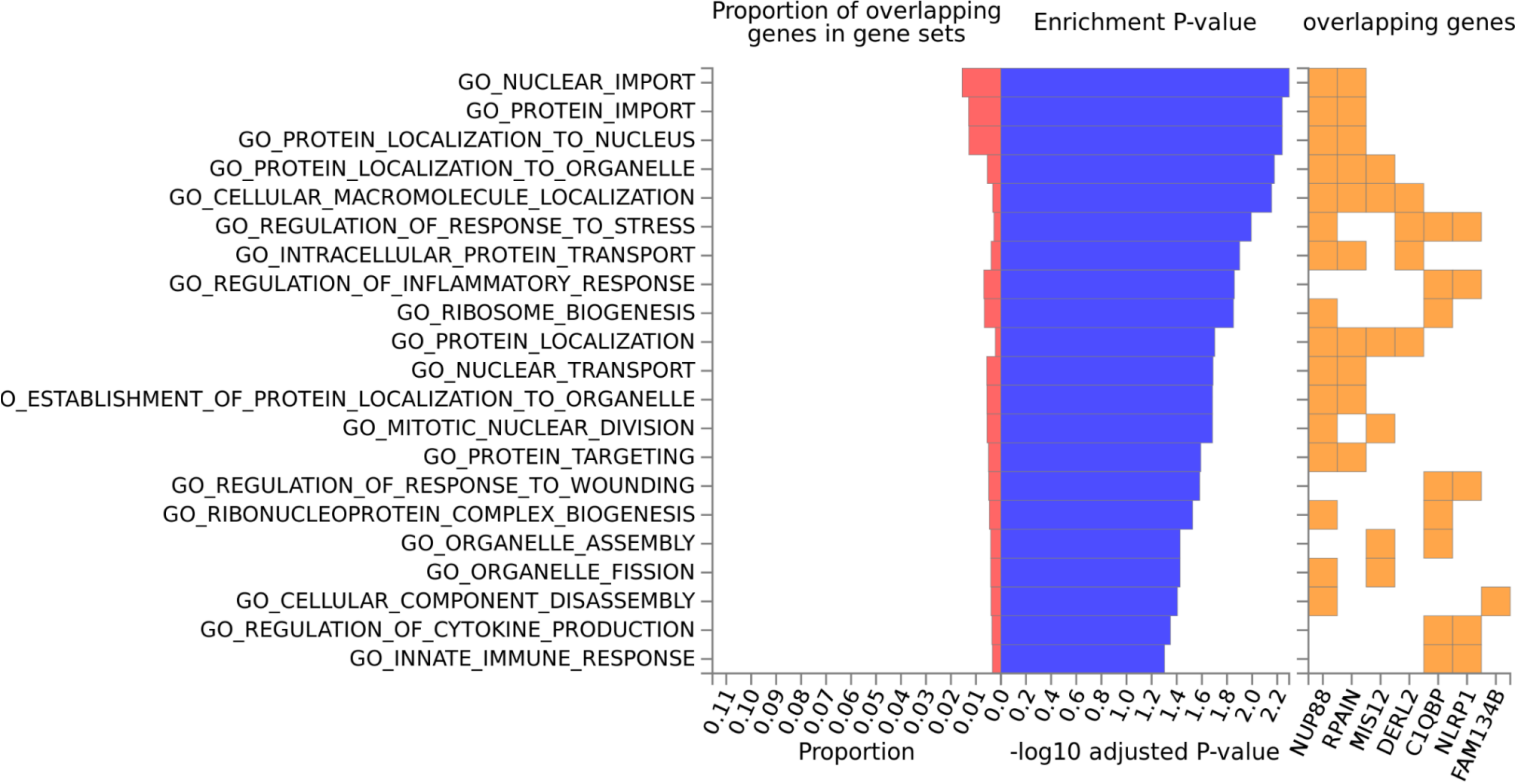
Pathway enrichment among genes implicated by position mapping for variants associated with ALT elevation case control status (p < 1 × 10^− 5^)

In addition, pathways enrichment analysis using MAGMA uses the entire p-value distribution and over-representation analysis implemented in GENE2FUNC of FUMA uses only the genes implicated by position mapping among the top associated variants (*p* < 1 × 10^− 5^). Many pathways in the inflammatory processes (Supplemental Tables 4 and 5 and Fig. 3) were implicated. In particular, innate immune response and regulation of cytokine production (implicated by *C1QBP* and *NLRP1*) were enriched (adjusted p-value < = 0.05) among genes implicated by these top associated variants. rs3865350 is an sQTL variant for *C1QBP* in fibroblast and lymphocytes (Supplemental Fig. 5), which encodes complement C1q binding protein. It regulates the transcriptional activity of hypoxanthine catabolic enzyme xanthine dehydrogenase (XDH) in cancer cells presumably by regulating the mRNA level of XDH transcriptional stimulators IL-6, TNF-α, and IFN-γ, and promotes the catabolism of hypoxanthine [28]. XDH also mediates reactive oxygen species (ROS) generation and apoptosis. Both innate immune response and oxidative stress are hypothesized mechanisms for DILI. It is worth to mention in the in vitro challenging/cloning experiment also implicated the role of adaptive response in atabecestat liver injury where CD4 + T-cell clones activated by the atabecestat metabolite DIAT were detected in five out of eight patients, and CD4 + and CD8 + clones activated by atabecestat were also detected although with a lower cloning efficiency [20]. These clones proliferated and secreted IFN-γ and IL-13 following atabecestat or DIAT stimulation [20].

It is also of interest that several eQTL/sQTL relationships between chr17 variants (rs3865350 and kgp8586964) and *NLRP1* and *C1QBP* were detected in fibroblast cells. Hepatic stellate cells (HSCs) account for 15% of total resident cells in the normal human liver and are resident mesenchymal cells with features of resident fibroblasts and pericytes. HSCs play key roles in liver fibrosis. HSCs are quiescent in the normal liver and become activated during liver injury, resulting in inflammation and activation of the innate immune system leading to liver fibrosis [29]. On the other hand, transient fibrosis could be a protective mechanism by maintaining cell attachment and the architecture of liver tissue via production of extracellular matrix proteins and a regenerative mechanism by repairing liver damage by producing growth factors [30, 31].

In the continuous trait analysis, we identified intronic variants in GABRA3 associated with max(ALT/ULN) reaching study wide significance threshold. GABRA3 in liver plays a protective role for DILI as GABRA3 is involved in altered signaling for driving hepatocyte proliferation to restore damaged hepatoctyes [32].

With the limited sample size, the suggestive GWAS signals implicate the potential role of inflammation in the atabecestat induced liver enzyme elevation. There are several limitations for this study besides the sample size. First, samples were genotyped in 4 batches (in particular, the last two samples were singleton samples, and there is no convenient way of assessing genotyping quality); second, due to the sample size limitation, the case control analysis used all samples and Fisher’s Exact test was used for association analysis (to handle the risk of potential cell count less than five in the 2 × 2 table used for statistical test), although the quantitative trait max(ALT/ULN) corrected for 5 principal components (PCs). We however cross-checked the top association results to provide additional supporting evidence and performed sensitivity analyses using logistic regression correcting for 2 PCs (running the risk of small cell count) and performing similar analysis in the population of European ancestry alone (with even smaller sample size); third, the phenotypes used is a risk factor for liver hepatotoxicity and not DILI, which is a more extreme phenotype and could help to uncover the genetic cause better; fourth, given the small sample size, the model did not explicitly correct for age and gender to use up another 2 degrees of freedom, even though the extent of ALT elevation was expressed as ALT/ULN and ULN is age and gender dependent. Clinical trials on several compounds with similar mechanism of action including lanabecestat and atabecestat (but not verubecestat) had revealed similar issues of liver enzyme elevation [33, 34]. It will be of interest to learn if there are overlapping mechanisms underlying the observed liver enzyme elevation. Future endeavors of this line of work may also encompass longitudinal repeat measurement modeling and/or use a different aggregation method other than maximum.

## Conclusions

We performed GWAS to identify genetic risk factors for atabecestat induced ALT elevation. The suggestive GWAS signals implicate the potential role of inflammation in the atabecestat induced liver enzyme elevation.

## Methods

### Clinical cohorts

The samples included in the analysis were pooled from three studies sponsored by Janssen Research & Development, LLC including 54861911ALZ2002 (NCT02260674), a Phase 2a study to evaluate the safety and tolerability of atabecestat (JNJ-54,861,911), an oral BACE1 inhibitor in participants with early Alzheimer’s Disease [17]; 54861911ALZ2004 (NCT02406027, terminated), the long-term extension study of (JNJ-54,861,911) in early Alzheimer’s disease spectrum patients [17]; and 54861911AZ2003(NCT02569398, terminated), Phase 2b efficacy and safety study of Atabecestat in participants who are asymptomatic at risk for developing Alzheimer’s Dementia (EARLY) [18]. In 54861911ALZ2002 and 54861911ALZ2004, 12 of 104 subjects (11.5%) experienced liver enzyme elevation, resulting in dosage modification and increased frequency of safety monitoring, but no case met the Hy’s law criteria (ALT or aspartate aminotransferase (AST) > 3× ULN and total bilirubin > 2× ULN) [17] to predict serious hepatotoxicity. Treatment discontinuation normalized ALT or AST in all except one with pretreatment elevation [17]. In 54861911ALZ2003, 40 of 371 subjects (10.8%) receiving atabecestat and 1 subject in the placebo from 54861911ALZ2003 experienced ALT or AST elevation (> 3x ULN), among which one subject met the Hy’s law criteria [18]. As a result, 54861911ALZ2003 and 54861911ALZ2004 were terminated early. Samples included in this genetic analysis were from subjects treated with atabecestat before the decision for trial termination and therefore not all cases with liver enzyme elevation were included. The clinical studies were carried out in accordance with the ethical principles outlined in the Declaration of Helsinki, Good Clinical Practices guidelines, and applicable regulatory requirements.

### Phenotype definitions

Two phenotype definitions for ALT elevation were used. First, a dichotomized ALT elevation case control status phenotype was used where cases were defined as having ALT level > = 3x ULN at any time during the study after atabecestat exposure, while controls were defined as having ALT < = 1x ULN at all times; Second, a continuous phenotype was used using log transformation of maximal ALT value (expressed as times the ULN) at any time during the study to approximate normal distribution. In total 42 cases and 141 controls were included in the GWAS using the dichotomized phenotype, while 285 samples were included in the GWAS using the continuous phenotype. A full list of case by ULN was provided in Supplemental Table 7.

### Genotyping

A total of 380 samples were genotyped using Omni2.5MExome or Omni2.5 M chips over 4 batches (2 of them were singleton presumably with samples from other studies by the genotyping vendor) at three facilities (Supplemental Table 8).

### Genotype data quality control

The genotype data were merged and QC’ed where samples exceeding 5% missingness rate were excluded, whereas SNP exceeding 5% missingness rate, deviating from Hardy-Weinberg equilibrium (*p* < 1 × 10^− 6^) or having minor allele frequency (MAF) below 1% were excluded. The total genotyping rate was 99.86%. Among the remaining participants, relatedness was assessed using pairwise identity by descent (IBD) estimation in PLINK [35]. Participants were excluded as needed to ensure that the estimated proportion of IBD (PI HAT) between any two remaining individuals was less than 0.3. Additionally, subjects (if any) with genetically inferred gender discrepancies from the gender provided in the clinical database were excluded. No subject was excluded in this study due to this reason. No outlier removal was applied to remove subjects 6 sigma away using EIGENSTRAT [36, 37] to maximize the power of the study. After genotyping QC, 374 samples were retained removing samples with low call rates and related samples (Supplemental Table 9).

The genotype data were further QC’ed using RICOPILI pipeline [38] with standard parameters [SNP-wise missingness rate 2%, Hardy-Weinberg equilibrium (*p* < 1 × 10^− 6^ in controls, or *p* < 1 × 10^− 10^ in cases)], except when sample-wise missingness rate was set to 4%.

### Genotype data imputation

To allow the exploration of a greater density of markers than what was genotyped directly, the imputation was performed using RICOPILI (minimac3 [39]/Eagle [40] v2.3.5) pipeline. Imputation of unobserved genotypes was based on the reference haplotypes from the 1,000 Genomes haplotypes; Phase 3 integrated variant set release in NCBI build 37 (hg19) coordinates from the 1000 Genome Project [41].

### Single marker GWAS analysis

Fisher exact test was used in the case-control association analysis without correcting for PCA using the directly genotyped markers passing SNP QC (n = 1,526,079). ~11 M imputed and directly genotyped markers were used in the max(ALT/ULN) genome wide association analysis. The continuous trait max(ALT/ULN) was analyzed in a linear regression model correcting for five principal components to account for population substructure and using the imputed genotype dosages. All association tests were performed using PLINK [35, 42]. Genotype quality was spot-checked post-GWAS to ensure the genotype quality where Genome Studio (Illumina, Inc.) Project files were available (Supplemental Fig. 3). A conventional genome-wide significance threshold of 5 × 10^− 8^ was used to declare study-wide significance. A list of variants with unadjusted p-value less than 1 × 10^− 5^ (Fisher’s exact test) or 1 × 10^− 6^ (linear regression) was also reported.

### GWAS sensitivity analysis

Fisher’s exact test cannot account for covariates. We also performed case-control logistic regression GWAS as a sensitivity analysis and additionally correct for two principal components. These analyses were also performed using RICOPILI pipeline [38] and the 1,000 Genomes haplotypes phase 3 reference panel. Additionally, the subset of homogenous samples of European ancestry were used as another sensitivity analysis for both case-control and continuous max(ALT/ULN) outcomes. These results were only used for cross-reference purpose only to ensure that population stratification was not the reason for the observed association signals.

### Multi-marker analysis of genomic annotation (MAGMA) gene-based analysis, gene set-based analysis, and variant annotations

Variant clumping to identify independent genomic locus and annotation was performed using FUMA [43]. In addition to single-marker-based GWAS, gene-based analyses followed by pathway enrichment analysis were computed using MAGMA (v1.07) [44] based on GWAS summary statistics. SNPs were mapped to 18,035 protein-coding genes (Ensembl v92). Genome-wide significance was defined at p = 0.05/18,035 = 2.77 × 10^− 6^. The MAGMA analyses were performed using FUMA [43].

In addition to MAGMA gene set based enrichment analysis, which uses the entire p-value distribution, gene set enrichment analysis was performed using variants with association p-value less than 1 × 10^− 5^ using GENE2FUNC feature of FUMA. Variants were annotated using FUMA using position-based, eQTL-based, and 3D chromatin interaction information. The Manhattan plots, QQ plots, and circos plots were generated using FUMA (v1.3.4b) [43] available from https://fuma.ctglab.nl/.

### Electronic supplementary material

Below is the link to the electronic supplementary material.

Supplementary Material 1

Supplementary Material 2

## Data Availability

Due to the proprietary nature of the data and privacy obligation, the request for the data will be a considered on a case-by-case basis and a Data Transfer Agreement is required to ensure the research is consistent with the scope of informed consent and necessary precaution is taken to protect patient privacy. Data request shall be directed to QSL.

## Abbreviations

AD: Alzheimer’s disease
ALT: Alanine transaminase
AST: Aspartate aminotransferase
DILI: Drug-induced liver injury
GWAS: Genome wide association study
ULN: Upper limit of normal
