# Supplementary material for: Genome-wide association study of abnormal elevation of ALT in patients exposed to atabecestat
Source: BMC Genomics. 2023 Sep 1;24:513. doi: 10.1186/s12864-023-09625-6 (PMC10472559; doi:10.1186/s12864-023-09625-6)
Supplement: Supplementary file 1 — Supplementary Material 1 [file 12864_2023_9625_MOESM1_ESM.docx]

***Supplemental Information***

**Supplemental Table 7**. Breakdown of ALT elevation by study and by the extent of elevation (including samples not genotyped)

**Supplemental Table 8**. Sample genotyping batch

**Supplemental Table 9**. Sample attritions from sample-level quality control steps

**Supplemental Figure 1**. Manhattan plots for single-variant association analyses (A) continuous and (B) dichotomized trait and gene-level association analyses (C) continuous and (D) dichotomized trait

**Supplemental Figure 2**. Q-Q plots for single-variant association analyses (A) continuous and (B) dichotomized trait and gene-level association analyses (C) continuous and (D) dichotomized trait

**Supplemental Figure 3**. Genotype cluster plots for the top associated variants

**Supplemental Figure 4**. eQTL relationship between rs8067359 and *NLRP1*

**Supplemental Figure 5**. sQTL relationship between rs3865350 and *C1QBP* (<https://gtexportal.org/home/snp/rs3865350>)

**Supplemental Table 7**. Breakdown of ALT elevation by study and by the extent of elevation (including samples not genotyped)

**Supplemental Table 8**. Sample genotyping batch

| **Batch** | **Batch Name** | **Sample size** | **Genotyping facility** |
| --- | --- | --- | --- |
| 1 | JnJ_Li_Omni25Exome_2018.01_gtReport_File | 188 (186) | Illumina |
| 2 | DILI_FinalReport/A3819 | 192 | Eurofins Scientific |
| 3 | 1148_16845_Omni25-8_FinalReport_Forward.txt | 1 | Covance Genomics Lab (CGL) |
| 4 | 1148_16740_Omni2.5_FinalReport_ForwardStrand.txt | 1 | Covance Genomics Lab (CGL) |
|  | Subtotal | 380 |  |

**Supplemental Table 9**. Sample attritions from sample-level quality control steps

| **Step** | **QC Measure** | **Sample size** |
| --- | --- | --- |
| 1 | Removed samples with low call rate (n=3) | 377 |
| 2 | Removed samples with genetically inferred gender discrepant from CRF gender (n=0) | 377 |
| 3 | Removed related or identical samples (n=3) | 374 |

**Supplemental Figure 1**. Manhattan plots for single-variant association analyses (A) continuous and (B) dichotomized trait and gene-level association analyses (C) continuous and (D) dichotomized trait

(A)


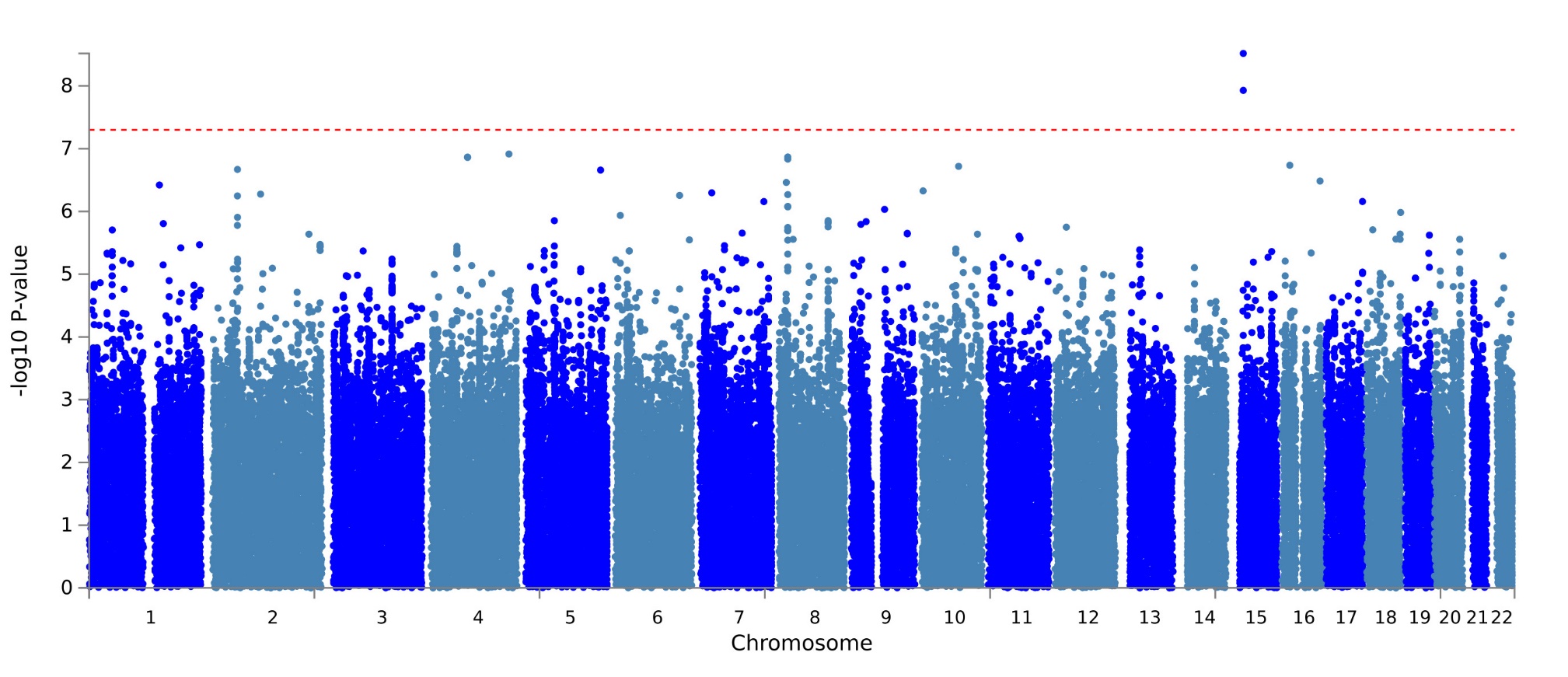


(B)


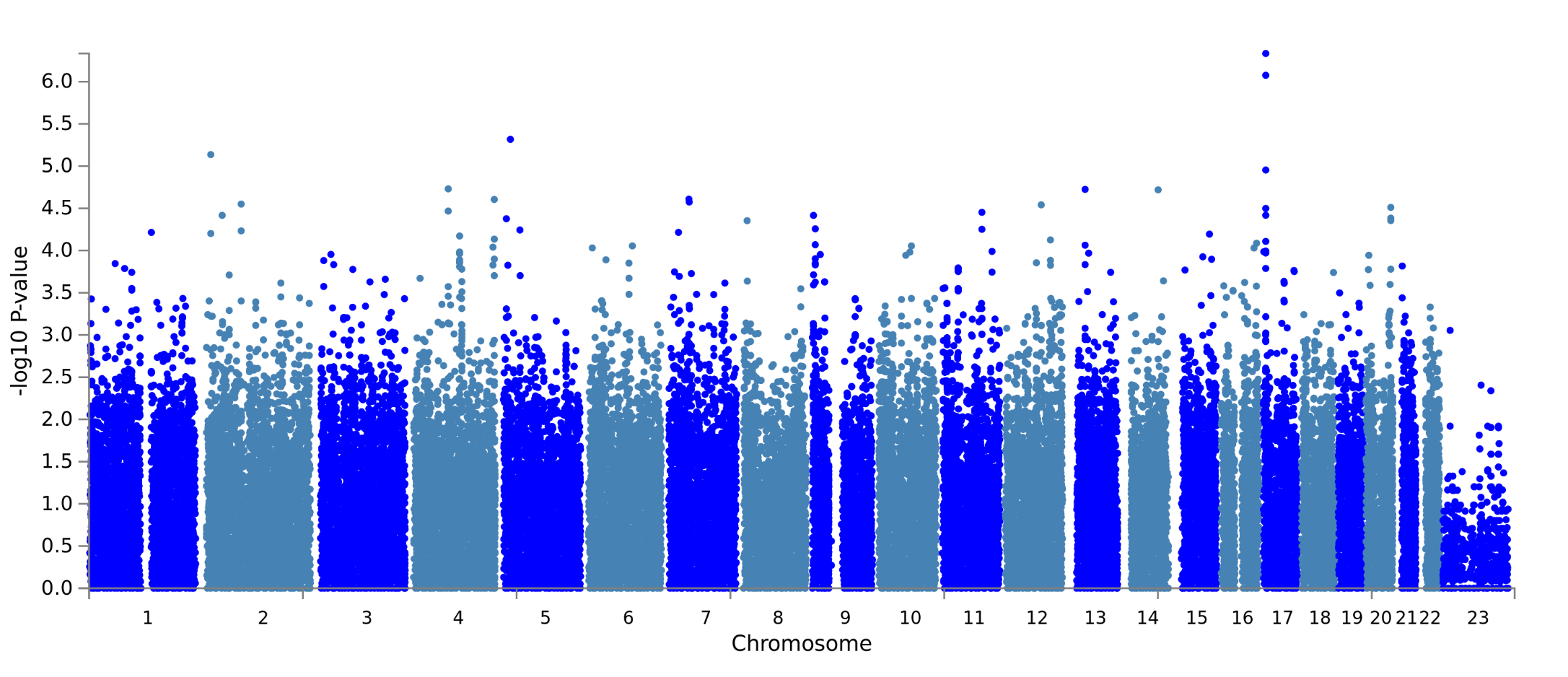


(C)


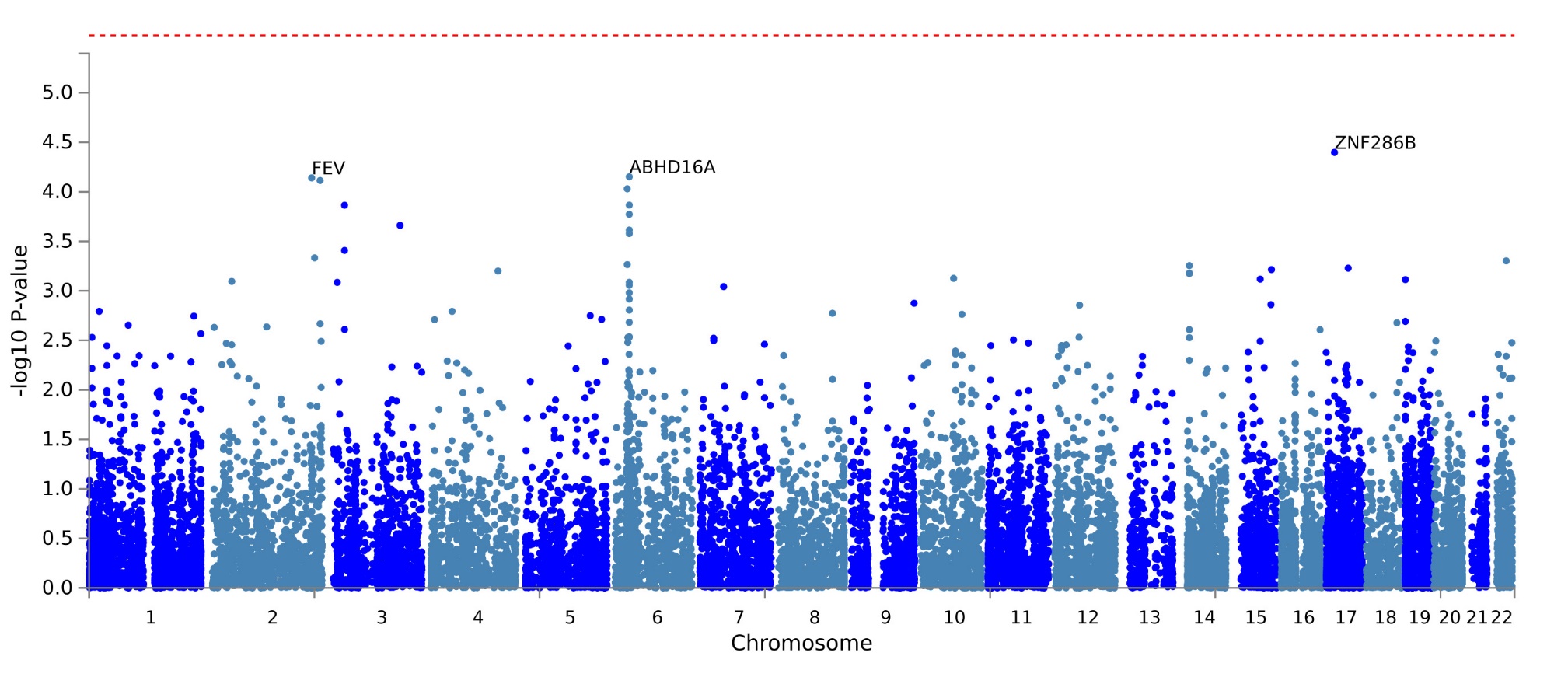


(D)


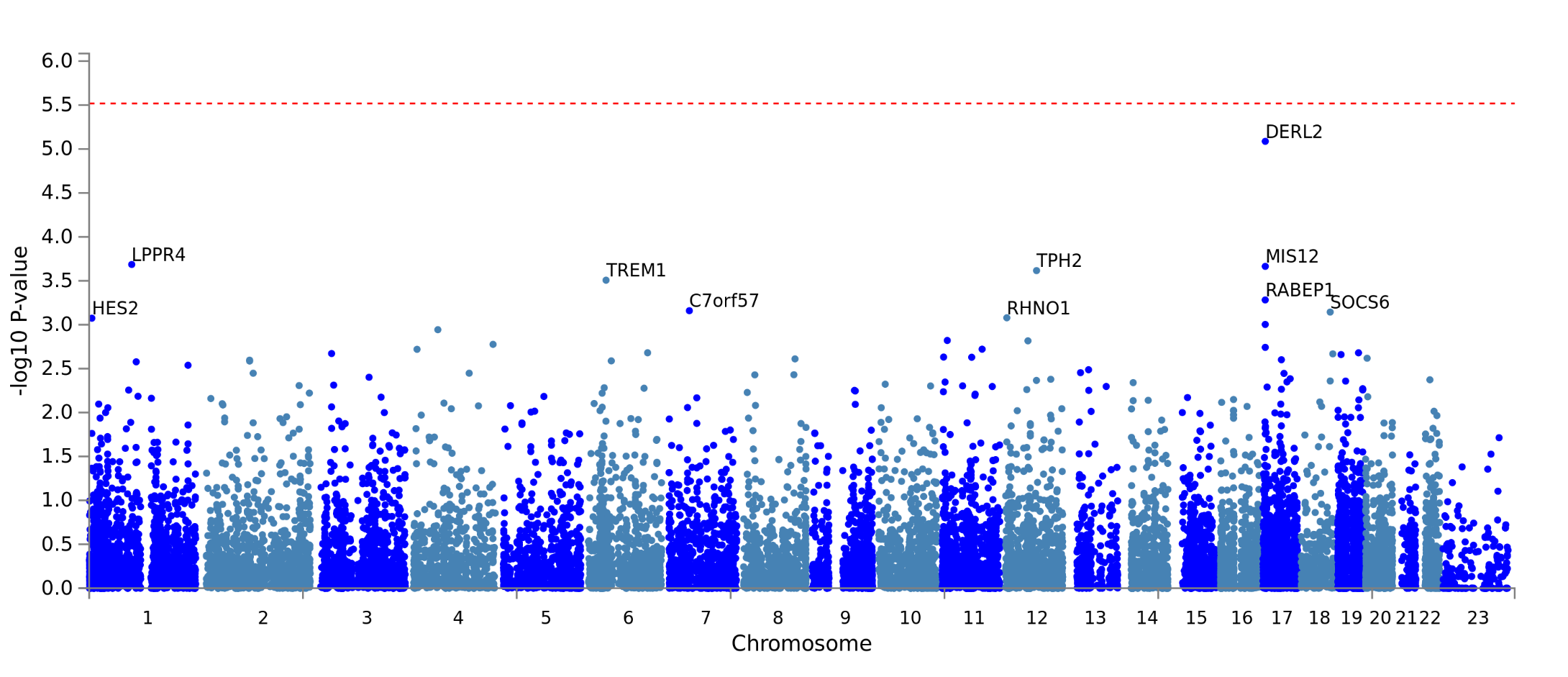


**Supplemental Figure 2**. Q-Q plots for single-variant association analyses (A) continuous and (B) dichotomized trait and gene-level association analyses (C) continuous and (D) dichotomized trait

(A)


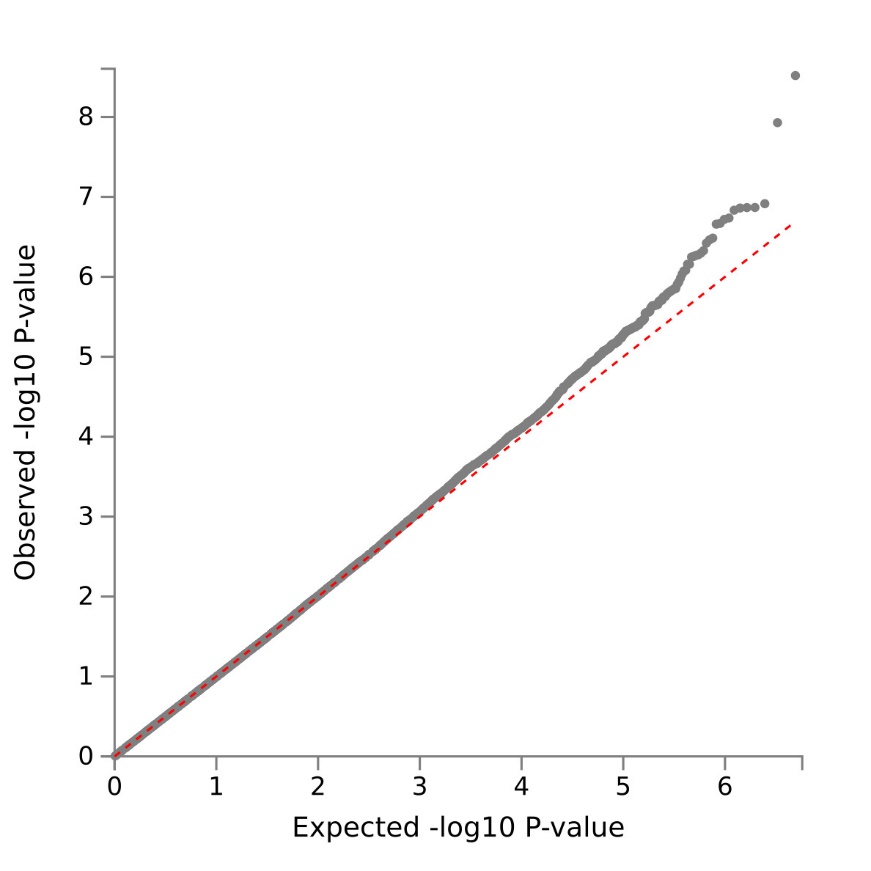


(B)


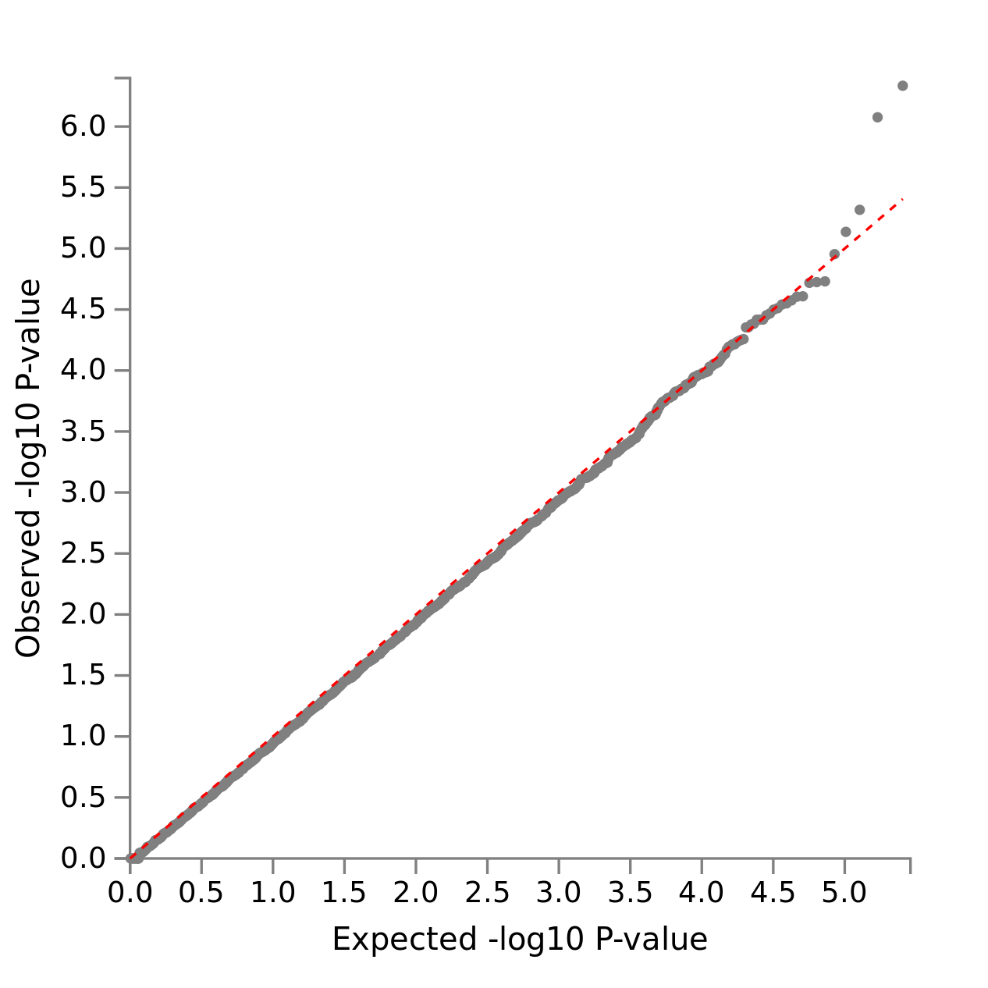


(C)


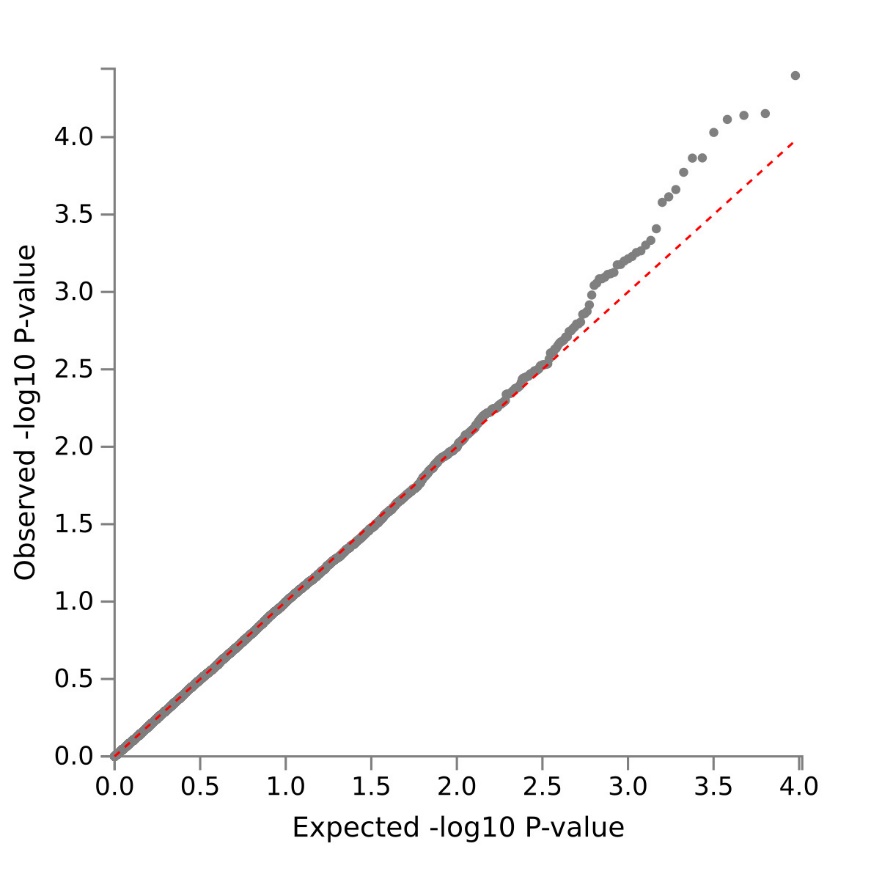


(D)


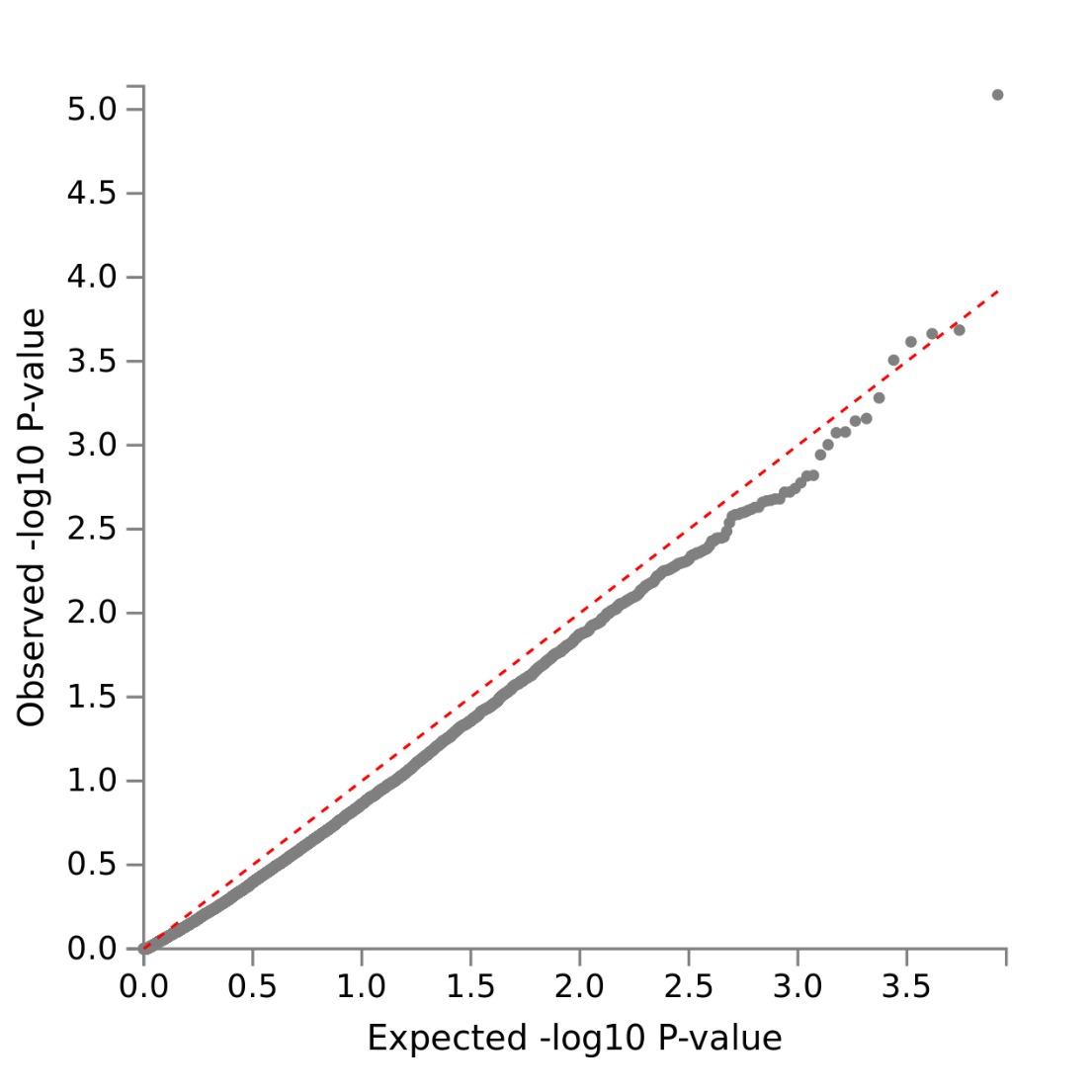


**Supplemental Figure 3**. Genotype cluster plots for the top associated variants

1. JnJ_Li_Omni25Exome_2018.01 batch

1. A3819

1. 1148_16845_Omni25-8 batch

**Supplemental Figure 4**. eQTL relationship between rs8067359 and *NLRP1* (<https://gtexportal.org/home/snp/rs8067359>)

Single-Tissue eQTLs for chr17_5505777_G_T_b38

Data Source: GTEx Analysis Release V8 (dbGaP Accession phs000424.v8.p2)


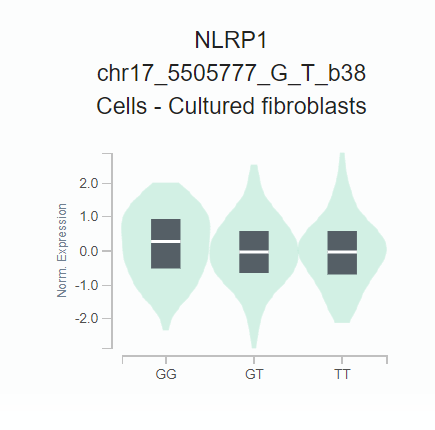


*p* = 2.2e-12

**Supplemental Figure 5**. sQTL relationship between rs3865350 and *C1QBP*

Single-Tissue sQTLs for chr17_5478547_C_T_b38

Data Source: GTEx Analysis Release V8 (dbGaP Accession phs000424.v8.p2)


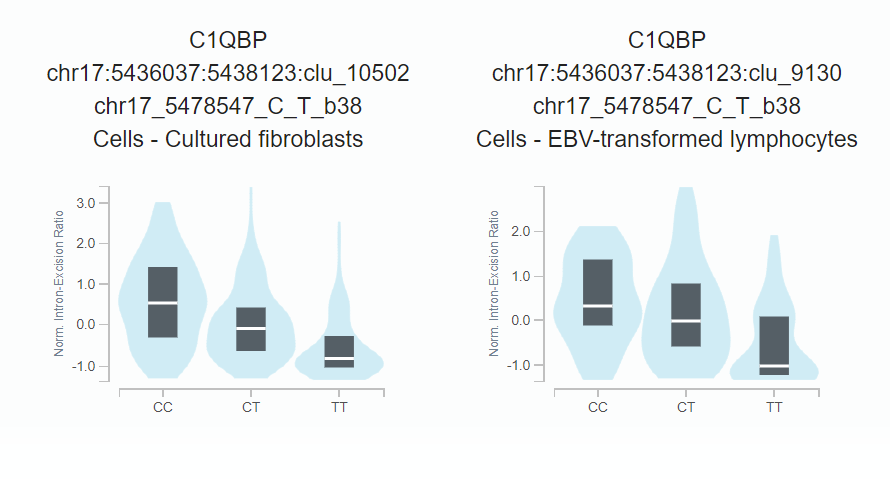


*p* = 7.0e-23 *p* = 2.2e-9
